# Infant leukaemia – faithful models, cell of origin and the niche

**DOI:** 10.1242/dmm.049189

**Published:** 2021-10-29

**Authors:** Alasdair Duguid, Domenico Mattiucci, Katrin Ottersbach

**Affiliations:** Centre for Regenerative Medicine, Institute for Regeneration and Repair, University of Edinburgh, 5 Little France Drive, Edinburgh EH16 4UU, UK

**Keywords:** Haematopoiesis, Niche, Infant leukaemia

## Abstract

For patients and their families, the diagnosis of infant leukaemia is devastating. This disease has not seen the improvements in outcomes experienced with other paediatric leukaemias and it is becoming ever more apparent that infant leukaemia is a distinct biological entity. Insights into some of the distinguishing features of infant leukaemia, such as a single mutation – the *MLL*-gene rearrangement, the biology of disease aggressiveness and lineage plasticity, and the high incidence of central nervous system involvement, are likely to be gained from understanding the interactions between leukaemic cells and their environment or niche. The origins of infant leukaemia lie in the embryonic haematopoietic system, which is characterised by shifting locations and dynamic changes in the microenvironment. Understanding this foetal or embryonic context is integral to understanding infant leukaemia development. Owing to its rarity and prenatal origins, developing accurate modelling systems for further investigation of infant leukaemia is essential. In this Review, we discuss how available *in vitro*, *ex vivo* and *in vivo* infant leukaemia models contribute to our current understanding of the leukaemia niche in embryonic development, established disease and specialised non-haematopoietic niches. The mechanistic insights provided by accurate models will help identify viable novel therapeutic options.

## Abstract (precis)

**Summary:** In this Review we discuss how *MLL* gene rearrangements cooperate with transient facilitators presented by the foetal environment to initiate the development of infant leukaemia, and evaluate current leukaemia models.

## Introduction

Leukaemias are the most common type of childhood cancers; however, the opposite is true in children under the age of 1 year (infants), where leukaemia is a relatively uncommon malignancy (Alfaar et al., 2017). Compared with childhood leukaemia, infant leukaemia differs in clinical presentation, cellular identity, genetic composition and prognosis. Infant leukaemia is a particularly challenging disease to treat. This is in part because of its rarity but also its poor response to conventional chemotherapy approaches and lack of novel treatments (Brown et al., 2019). Indeed, large-scale international collaborative trials have failed to improve outcomes for these patients; the recently reported INTERFANT-06 randomised trial had a negative result with overall survival rates of ∼50% (Pieters et al., 2019). The ability to explore new therapies is limited by a lack of biological understanding of the underlying disease processes. Infant leukaemia can present as lymphoid, myeloid or mixed-lineage and can even display lineage plasticity during treatment (Wölfl et al., 2018). It is also associated with high rates of extramedullary disease, including involvement of the central nervous system (CNS). Up to 80% of lymphoid and 50% of myeloid infant leukaemia are associated with rearrangements of the gene encoding lysine methyltransferase 2A (*KMT2A*, also known as and hereafter referred to as *MLL*) that result in the fusion of *MLL* to another gene (Harrison et al., 2010; Hilden, 2006). A variety of fusion partners have been reported, with the transcriptional regulator AF4/FMR2 family member 1 (*AFF1*, hereafter referred to as *AF4*) being the most common in lymphoid infant leukaemia. The resulting MLL-AF4 fusion protein is produced by a characteristic t(4;11) chromosomal translocation. As sequencing studies have failed to identify co-driver mutations, this single gene rearrangement appears to be sufficient to induce and maintain the development of leukaemia (Agraz-Doblas et al., 2019; Andersson et al., 2015). However, this is quite unlike the genetic basis of cancers in adults, where an accumulation of mutations is usually required for malignant transformation.

Several lines of evidence have led to a focus on the foetal origins of infant leukaemia. The extremely high concordance rates of infant leukaemia in monozygotic twins have been known for many decades. Although clearly a rare event, the concordance rates are consistently reported as approaching 100% (Greaves et al., 2003). To gather information about the mutational landscape at a very early time point in the post-natal life of children who subsequently developed MLL-AF4 infant leukaemia, Gale and colleagues retrospectively analysed routine blood spot screening tests, which were taken hours after birth, and detected the leukaemia-specific *MLL-AF4* fusion in these children (Gale et al., 1997). Taken together, the data obtained from twins and newborn blood spots provide compelling evidence for an *in utero* origin for infant leukaemia.

Recent experimental data point towards leukaemia initiation within the foetal liver (Barrett et al., 2016). Consequently, attention has been given to understanding this foetal environment, or ‘niche’, and how it contributes to leukaemogenesis. Analysis of many adult cancers has shown that the environment in which cancer stem cells reside, i.e. the cancer niche, is of critical importance for survival and maintenance. Indeed, cancer stem cells create these cancer niches by reprogramming surrounding stromal cells to contribute towards cell-death resistance, evasion of immune responses, replicative immortality and metastatic potential (Plaks et al., 2015). This specialised microenvironment has been explored in adult leukaemias, where the bone marrow acts as the niche. Remodelling of the bone marrow stroma occurs in order to support leukaemia cells and suppress normal haematopoiesis through bi-directional cell-cell interactions, production of soluble factors and immune escape via T cell inhibition (Krause and Scadden, 2015; Oh et al., 2019; Schepers et al., 2015). This protective environment poses a major challenge to successfully eradicating the leukaemia stem cell, an essential step in providing long-term disease-free remission. There is also evidence that, under certain circumstances, the bone marrow niche may provide a permissive environment for leukaemia initiation (Wiseman, 2011). It is, therefore, reasonable to assume that the developing haematopoietic niche, in particular the foetal liver, plays a role in the *in utero* pathogenesis of infant leukaemia. Furthermore, when considering experimental model systems of infant leukaemia, replicating this foetal context during disease development is an important consideration.

This Review summarises the haematopoietic niche during embryogenesis, discusses current understanding of infant leukaemia development in this context and considers the model systems available to investigate this further.

## The developmental haematopoietic niche

Blood development in vertebrates is highly conserved and has been extensively studied in zebrafish (Konantz, et al., 2019) and in mouse. Current evidence supports the existence of three distinct waves of haematopoietic cell emergence. These waves involve sequential, spatially and temporally separated niches within the embryo, each featuring a complex interplay between haematopoietic cells and their environment. There have been several detailed reviews on this topic (Dzierzak and Bigas, 2018; Mirshekar-Syahkal et al., 2014; Pinho and Frenette, 2019).

The ‘first wave’ of blood production occurs in the yolk sac – an extraembryonic niche – starting at embryonic day (E)7 in the mouse. It involves the formation of blood islands composed of vascular endothelial cells that surround primitive erythroid cells, megakaryocytes and macrophages (Palis et al., 1999; Tober et al., 2007). These early cells address the immediate needs of the embryo, in particular to support tissue oxygenation in conjunction with the developing circulatory system. Lineage tracing studies have demonstrated that contribution from this wave of haematopoiesis to long-term definitive blood production is minimal (Busch et al., 2015).

Evidence of definitive haematopoiesis has been found during the ‘second wave’ of blood production starting at E8.5, also originating in the yolk sac and mainly being composed of erythro-myeloid progenitor cells (Frame et al., 2013). It is, however, only during the ‘third wave’ beginning at E10.5, that the first fully functional haematopoietic stem cells (HSCs) are formed. A specialised subset of dorsal aortic endothelial cells – the haemogenic endothelium, located within the aorta-gonad-mesonephros (AGM) region – undergoes a transition to become HSCs capable of long-term multilineage repopulation upon transplantation into adult recipients (de Bruijn, 2000; Kissa and Herbomel, 2010; Medvinsky and Dzierzak, 1996; Zovein et al., 2008). Regulation of the endothelial-to-haematopoietic transition is complex, involving the key transcription factors Runx1 and Gata2 as well as the Notch pathway signalling (Ottersbach, 2019). This transition is embedded in a dynamic, multi-cellular niche that contributes to the process through cell-cell interactions and the secretion of soluble factors. HSCs have also been detected in the yolk sac and the placenta; but whether they are generated there is currently unclear (Gekas et al., 2005; Ottersbach and Dzierzak, 2005; Robin et al., 2009). There is, however, evidence for limited HSC generation in the vasculature of the embryonic head (Li et al., 2012), suggesting that these cells can be generated outside the AGM.

The next stage of blood development, when HSCs migrate from the AGM to colonise the foetal liver, is of particular interest to those studying infant leukaemia. From E11 onwards, there is rapid HSC expansion, with highly proliferative HSCs found in this new niche (Bowie et al., 2006). Within the enlarging pool of HSCs differentiation begins to occur, and the full haematopoietic system is established. This phase of rapid expansion and differentiation appears to be important in the development of infant leukaemia. Our group has previously demonstrated this in the Mll-AF4^+^ VEC-Cre^+^ conditional mouse model of infant B-cell leukaemia, which was developed by targeting the first definitive HSCs with the *Mll-AF4* fusion gene by using a VE-cadherin-Cre recombinase (Barrett et al., 2016). Expression of the fusion gene induced a ‘pre-leukaemia phase’ in the foetal liver between E12 and E14. Although the malignant transformation was incomplete, we observed increased HSC activity and self-renewal, B-lymphoid bias and expansion of pro-B lymphocytes during this restricted developmental window. Importantly, these features were absent in other developmental haematopoietic niches, the yolk sac and AGM (Barrett et al., 2016). Our findings suggest that, during this stage of development, the foetal liver niche contributes to the initiation of MLL-AF4 infant leukaemia, a concept that will be discussed in more detail later in this Review.

**Fig. 1.**
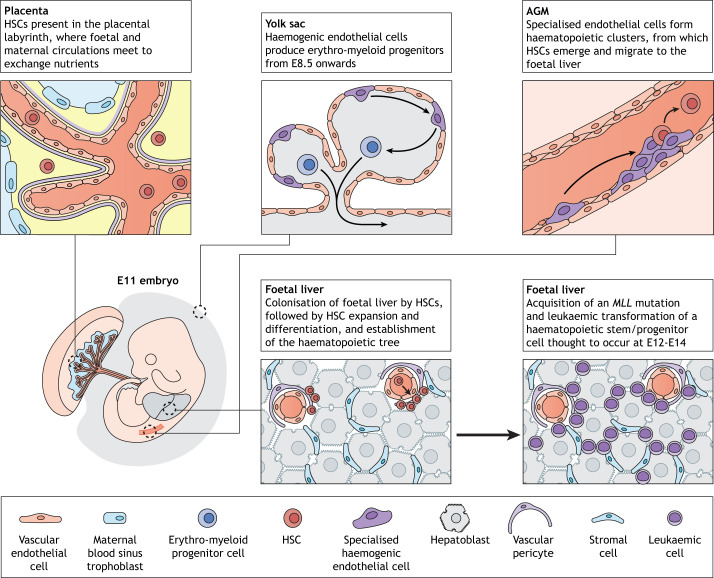
**Haematopoietic niches in the developing embryo at E11.** *Placenta*: HSCs expand within the placental labyrinth, where the foetal and maternal circulation meet to allow gas and nutrient exchange. It is still unclear whether these HSCs are produced in, or merely circulate through, this niche. *Yolk sac*: definitive haematopoiesis occurs in the yolk sac from E8.5 onwards. Haemogenic endothelial cells undergo endothelial-to-haematopoietic transition to produce erythro-myeloid precursors. AGM: specialised vascular endothelial cells in the dorsal aorta undergo an endothelial-to-haematopoietic transition, forming haematopoietic clusters. HSCs emerge from these clusters and migrate to the foetal liver. *Foetal liver*: HSCs seed this new niche through blood vessels and interact closely with the vascular endothelial cells of the portal veins and the Nestin^+^ Ng2^+^ pericytes within the foetal liver. Hepatoblasts and stromal cells also form part of the foetal liver-HSC niche. Here, HSCs undergo a phase of rapid expansion and differentiation, which forms the haematopoietic tree – a process thought to be hijacked in the development of infant leukaemia. As illustrated, multiple sites harbour and/or produce HSCs and other haematopoietic progenitors during this stage of embryonic development. However, only one of these niches, the foetal liver, can support leukaemic transformation, leading to infant leukaemia. This unique feature of the foetal liver strengthens the case of niche-specific factors playing a key role in the leukaemogenic process. AGM, aorta-gonad-mesonephros; HSC, haematopoietic stem cell.

Characterisation of the HSC niche in the foetal liver is not yet complete. Components that have so far been identified include pericytes associated with portal vessels and hepatoblasts, as illustrated in Fig. 1. Tamplin et al. performed high-resolution live imaging in a transgenic zebrafish line. Here, the definitive haematopoietic stem and progenitor cells (HSPCs) expressed a fluorochrome reporter driven by the Runx1+23 enhancer and could be followed when colonising the caudal haematopoietic tissue (CHT), i.e. the zebrafish equivalent of the foetal liver. Live tracking demonstrated remodelling of perivascular endothelial cells in the CHT upon arrival and anchoring of HSPCs by mesenchymal stromal cells. This arrangement of HSPCs and niche cells appears to be conserved in the mouse foetal liver (Tamplin et al., 2015). Indeed, the importance of perivascular cells in the murine foetal liver-HSC niche has been shown by Khan et al., where portal vessel-associated nestin- and chondroitin sulfate proteoglycan 4-positive (Nestin^+^ Ng2^+^) pericytes form an HSC niche in the foetal liver and are required to support HSC expansion *in vivo* (Khan et al., 2016).

Sugiyama et al. have highlighted the role of delta like non-canonical Notch ligand-positive (Dlk1^+^) hepatoblasts in supporting the proliferation of HSCs and the formation of erythroblastic islands necessary for the production of red blood cells within the foetal liver. Their results demonstrated that *Map2k4*-deficient mice, which lack hepatoblasts, show reduced HSC proliferation. Hepatoblasts were also found to be in close proximity to erythroblastic islands. Expression of erythropoietin, a hormone that stimulates the production of red blood cells, is restricted to Dlk1^+^ hepatoblasts, suggesting a role of this hepatoblast lineage specifically in supporting erythropoiesis within the foetal liver (Sugiyama et al., 2011).

The final step in haematopoietic development, the transition from foetal to adult haematopoiesis, represents the shift in HSCs from the foetal liver to the bone marrow just before birth, although this relocation starts a lot earlier in human foetuses (Ivanovs et al., 2017). This transition is guided by changes in adhesion molecule expression and chemoattractant signals, as the foetal liver switches from haematopoietic to hepatic function (Ciriza et al., 2013). Khan et al. found that the post-birth haemodynamic changes in the portal circulation following the cessation of umbilical blood flow are connected with cellular changes in the liver niche. Portal vessels transitioned from an arterial to venous phenotype with an associated loss of HSC-supportive Nestin^+^ pericytes by post-natal day 8. This transition coincided with a rapid reduction in liver HSCs (Khan et al., 2016). Coskun et al. have shown that this shift coincides with the vascularisation of bone marrow, reduction in HSC proliferation and the establishment of long-term HSCs (LT-HSCs), which depends on bone marrow niche osteolineage cells (Coşkun et al., 2014). Thorough understanding of haematopoietic niche development and the factors that affect it is essential in identifying the processes that can initiate leukaemogenesis in the developing embryo.

## Aspects of niche involvement in infant leukaemia

It is not possible to easily explore the foetal origins of infant leukaemia in humans due to the rarity of the condition and the obvious challenges in obtaining embryonic tissue. To develop model systems is, therefore, a real need – but generating representative experimental models is challenging. It requires replicating the transitions between multiple haematopoietic niches in embryonic development and expressing fusion oncogenes in specific cell types at precise developmental stages. As discussed in this section, exploration of model systems has shown us that *MLL*-rearranged leukaemia cells interact with the developmental niche, and that the developmental stage of the niche can influence the capacity for malignant transformation and leukaemia lineage determination.

### Interactions between leukaemic cells and the niche

*In vitro* models derived from murine and human stem cells engineered to express oncogenes are often used to study leukaemia development because these systems can be manipulated with relative ease. This gives the advantage of being able to replicate the correct cell with the correct mutation but lacks the ability to replicate the complexity of the niche. This is particularly true when studying diseases with an embryonic origin, like infant leukaemia, where the niche is continuously changing. The need for modelling niche-dependent disease processes is highlighted where *in vitro* models are unable to replicate certain disease features, and elements of the niche might be the missing piece of the puzzle. Interestingly, *in vitro* infant leukaemia models do not undergo malignant transformation, thus motivating researchers to closely examine the niche as potentially controlling disease initiation. Nevertheless, *in vitro* models for infant leukaemia have provided some important insights, which are summarised below.

Menendez et al. analysed bone marrow-derived mesenchymal stromal cells (BM-MSCs) for cytogenetic abnormalities across a spectrum of children with B-lymphoblastic leukaemia (B-ALL) (Menendez et al., 2009). They found that where leukaemia cells carried the *MLL-AF4* fusion, the same fusion was present in a subset of BM-MSCs. This finding was unique to t(4;11), as the BM-MSCs from all other assessed B-ALL cytogenetic subtypes remained cytogenetically unaltered. When comparing *MLL-AF4* fusion-harbouring leukaemia blasts and BM-MSCs, the authors also found that only the leukaemia blasts expressed monoclonal immunoglobulin gene rearrangements. This finding implies that the fusion arises in a shared precursor cell prior to haematopoietic differentiation. The *MLL-AF4* fusion has a strikingly different effect on haematopoietic cells compared with BM-MSCs. BM-MSCs virally transduced with *MLL-AF4* demonstrated normal homeostasis in culture, without any apparent proliferative advantage compared to untransduced or empty-vector controls (Menendez et al., 2009). These findings raise the question of whether cytogenetically abnormal BM-MSCs – that form part of the haematopoietic niche – and/or their foetal precursors have a role in leukaemia development and maintenance. The implication of this for modelling MLL-AF4 infant leukaemia is that understanding and replicating the cellular context in which the fusion oncogene initiates, most likely in pre-haematopoietic cells, may be required to generate a truly representative disease model.

Building on this, Bueno et al. aimed to develop an *in vitro* model of infant leukaemia by using human embryonic stem cells (hESCs) and hESC-derived haematopoietic cells to provide a cellular context during early developmental stages (Bueno et al., 2012). The hESCs were transduced with *MLL-AF4* and subjected to embryoid body differentiation to mimic early developmental events and, therefore, to assess early haemogenic specification. *MLL-AF4* expression was not sufficient for leukaemia transformation *in vitro* or *in vivo* when these cells were transplanted into immunodeficient mice. There was an increased specification of haemogenic precursors although, further downstream, differentiation favoured an endothelial rather than haematopoietic fate, supporting the theory that MLL-AF4 subverts normal haematopoiesis. In a subsequent study, the same group used a similar technique to develop a cellular model using expression of both *MLL-AF4* and its reciprocal fusion gene *AF4-MLL* in hESCs (Bueno et al., 2019). This design reflects the observation that 50% of patients suffering from B-ALL with t(4;11) express this reciprocal fusion gene. The two fusion oncogenes cooperate, which results in increased output of haemogenic cells as well as both haematopoietic and endothelial primed cells. But, again, there was no evidence of malignant transformation in this model, suggesting that the niche microenvironment is, indeed, essential in infant leukaemogenesis.

Böiers et al. created an *in vitro* cellular system to investigate ETV6-RUNX1 childhood B-ALL leukaemia (Böiers et al., 2018). Even though the disease occurs later in life than infant leukaemia, there is strong evidence that childhood B-ALL leukaemia-initiating mutations also occur *in utero*. The authors used genome-engineered human pluripotent stem cells to express the ETV6-RUNX1 fusion protein and focused on a developmentally restricted CD19^−^ IL7R^+^ progenitor that was hypothesised to be the B-ALL cell of origin. They reported a pre-leukaemic phenotype with partial B-cell maturation block and co-expression of myeloid markers. As in the models developed by Bueno et al., malignant transformation does not occur in this cellular system, highlighting the limitations of *in vitro* modelling where potentially significant factors, including developmental context and niche-dependent environmental cues, cannot be easily mimicked (Böiers et al., 2018).

Another study by Wei and colleagues employed a murine xenograft model expressing the *MLL*-*AF9* fusion gene (*AF9*, officially known as *MLLT3*) to demonstrate how niche-dependent processes affect the determination of lineage in leukaemia (Wei et al., 2008). This *MLL*-*AF9* fusion oncogene is common in both infant and adult leukaemias and, in infants, can result in a myeloid- or lymphoid-lineage acute leukaemia. Wei and colleagues transduced human CD34^+^ cord blood cells with *MLL-AF9* and transplanted these cells into three immunodeficient adult recipients with different bone marrow microenvironmental conditions. Expression of the fusion transgene triggered malignant transformation of the transduced cells, as NOD/SCID and β_2_-microglobulin-null NOD/SCID recipients developed myeloid, B-cell lymphoid and mixed-lineage acute leukaemias (Wei et al., 2008). Transplanting the cells in NS-SGM3 mice – a NOD/SCID strain transgenic for three human cytokines that are known to regulate haematopoiesis (Feuring-Buske et al., 2003) – resulted exclusively in acute myeloid leukaemia (AML). These results demonstrate that leukaemia lineage determination can be influenced by the microenvironment, as exemplified by the expression of human cytokines in the NS-SGM3 recipients (Wei et al., 2008). A significant limitation in studying the leukaemia niche with these xenograft models is the species mismatch between the human leukaemia and the murine niche, a feature that is partially overcome in the NS-SGM3 mice. Although these mice express human cytokines in a non-physiological manner, they partly correct the species mismatch and show how manipulating microenvironmental cues can affect leukaemia lineage. Thus, although *in vitro* models have helped identify potential cells of origin and have allowed researchers to study the direct effects of oncogenes on target cells, successful leukaemic transformation of transduced HSPCs appears to require signals from the niche that are not present in *in vitro* models.

### Developmental stage of the niche and malignant transformation

An important consideration in leukaemia modelling is initiating the disease in the appropriate cell of origin. The infant leukaemia cell of origin is unknown, and the hunt for it has been limited by our incomplete understanding of lymphoid and myeloid development in the foetus. Recently, Popescu et al. and Ranzoni et al. have employed single-cell sequencing approaches to provide high-resolution details on human foetal liver haematopoiesis and foetal HSC differentiation as well as on lineage commitment trajectories, respectively (Popescu et al., 2019; Ranzoni et al., 2021). These are extremely valuable haematopoietic blueprints to reference against when homing in on the infant leukaemia cell of origin. In the same vein, O'Byrne et al. carried out detailed immunophenotypic, single-cell transcriptomic and functional analyses of human foetal B-cell development to describe a foetal B-cell hierarchy that is distinct from that of adults (O'Byrne et al., 2019). Particularly, the authors identified a newly described CD10^−^ PrePro-B-progenitor as the earliest B-lineage-restricted progenitor emerging in the foetal liver. Upon colonisation of foetal bone marrow, there is a massive expansion of B-lymphopoiesis with Pre-Pro-B-progenitors present at high frequency. By contrast, stable low-level production of B-cell progenitors was observed in matched foetal liver samples. These differences suggest that niche-dependent regulatory processes govern early B-lymphopoiesis. O'Byrne et al. also demonstrated that this novel CD10^−^ PrePro-B-progenitor shares transcriptional similarities with infant B-ALL blasts, identifying it as a potential cell of origin.

**Fig. 2.**
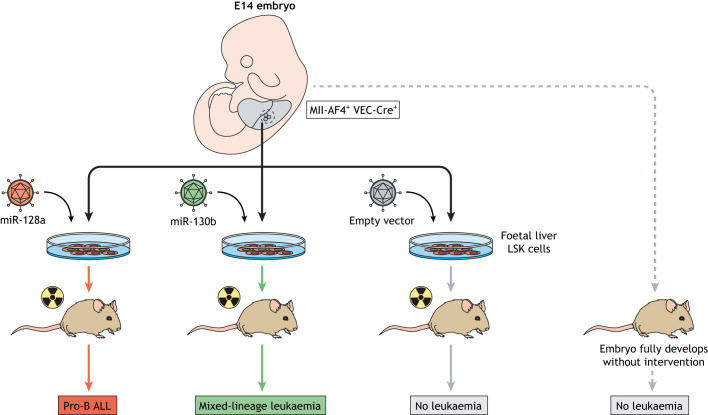
**Mll-AF4^+^ VEC-Cre^+^ foetal liver Lineage^−^Sca1^+^cKit^+^ (LSK) cells undergo leukaemia transformation upon overexpression of specific miRNAs.** As described previously, the Mll-AF4^+^ VEC-Cre^+^ conditional mouse model does not develop leukaemia on its own (Barrett et al., 2016). However, leukaemic transformation of foetal liver LSK cells from this mouse strain is possible during the pre-leukaemic window at E14, when overexpressing miR-128a and miR-130b that are known to be upregulated in infant leukaemia patients but not in this strain. Importantly, overexpression of miR-128a and miR-130b triggers different leukaemia lineages, thereby providing models for the range of phenotypes seen in MLL-AF4-driven infant leukaemia patients. Pro-B ALL, pro-B cell acute lymphoblastic leukaemia; VEC-Cre, vascular-endothelial-cadherin promoter-driven Cre recombinase.

Our group, as discussed above, used a murine MLL-AF4 infant leukaemia model to induce a pre-leukaemia state during a limited developmental window between E12 and E14 (Barrett et al., 2016). Upon further investigation, this pre-leukaemic model led to identification of the foetal liver lymphoid-primed multipotent progenitor (LMPP) as a potential leukaemia cell of origin (Malouf and Ottersbach, 2018). However, targeting Mll-AF4 protein expression to LMPP cells did not achieve a full leukaemic phenotype, which was puzzling considering that MLL-AF4 on its own is sufficient to cause infant leukaemia in patients and hinted at possible species differences. Indeed, we recently resolved this by identifying two microRNAs (miRNAs), i.e. miR-128a and miR-130b, that are upregulated in human MLL-AF4 infant and paediatric leukaemia patient samples but not in the Mll-AF4^+^ VEC-Cre^+^ mouse model (Malouf et al., 2021). By overexpressing these miRNAs individually in foetal liver Lineage^−^Sca1^+^cKit^+^ (LSK) cells during the E12-14 pre-leukaemic window, we were able to induce miRNA-dependent lineage-specific acute leukaemias that accurately replicate the spectrum of MLL-AF4 infant leukaemia phenotypes (Fig. 2). Combined, these findings emphasise that the foetal liver niche during this narrow stage of development is a crucial component in the initiation of infant leukaemia. Niche-dependent factors in the foetal liver, unlike the developmentally earlier niches of AGM or yolk sac, appear to be key factors in mediating this leukaemic transformation.

### Determination of niche and leukaemia lineage

**Fig. 3.**
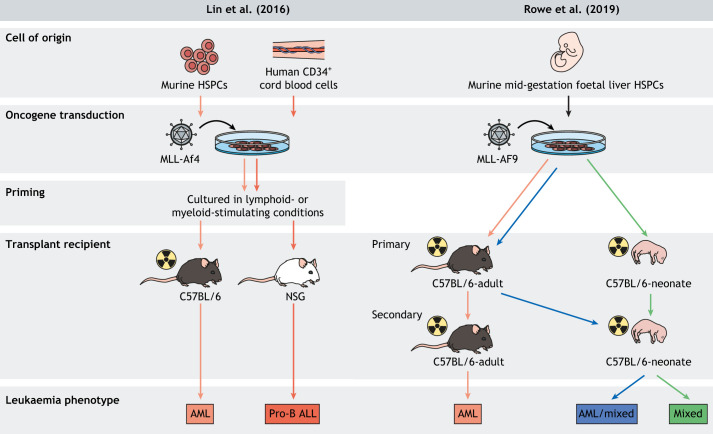
**Comparison between murine infant leukaemia models developed by Lin et al. (2016) and Rowe et al. (2019).** Lin et al. (left) used a high-titre retrovirus to introduce the chimeric human-murine *MLL-Af4* fusion transgene into both primary human cord blood cells and murine HPSCs. Transplantation of the transgenic cells in different hosts resulted in different leukaemia types despite being driven by the same fusion oncogene, highlighting the importance of species-specific differences. Rowe et al. (right) used retrovirus to transduce the human leukaemia fusion oncogene *MLL-AF9* into murine foetal liver HSPCs. By transplanting the transduced cells in mice of different ages, the authors investigated the effects of age on leukaemia lineage determination. AML, acute myeloid leukaemia; HSPC, haematopoietic stem and progenitor cell; NSG, non-obese diabetic/severe combined immunodeficiency/gamma; Pro-B ALL, pro-B cell acute lymphoblastic leukaemia.

Lin et al. have developed a murine model of infant leukaemia using high-titre retroviral transduction of the hybrid human-murine *MLL-Af4* fusion gene. *Af4* is the murine homolog to human *AF4*, a highly conserved *MLL* fusion partner (Lin et al., 2016). In that study, the authors transduced both murine HSPCs and human cord blood CD34^+^ cells, followed by transplantation into C57BL/6 and NSG mice, respectively. The ‘fully murine’ model developed myeloid leukaemia and the xenograft model developed B-cell lymphoid leukaemia. In addition to the inherent difference between the immune statuses of the transplant recipients, a possible explanation for the diverging leukaemia lineage determination is due to the different interactions of transduced murine and human cells with the murine bone marrow niche. To determine the influence of these microenvironmental signals, the transduced cells were cultured with either myeloid- or lymphoid-stimulating cytokines prior to transplant, as lineage ‘priming’ has been previously shown to influence the leukaemia phenotype (Wei et al., 2008). The resulting leukaemia had a similar phenotype and lineage, irrespective of culture conditions. A strong underlying lymphoid bias was seen when culturing human CD34^+^ cells expressing MLL-Af4 under myeloid conditions compared with cells expressing MLL-AF9. In MLL-Af4 cultures, a population of B-lymphoid CD19^+^ CD33^−^ cells persisted, whereas CD19^−^ CD33^+^ cells – despite their myeloid cell surface phenotype – continued to upregulate key B-lymphoid genes. On transplantation from these cultures, B-ALL developed even where a residual CD19^+^ B-lymphoid cell population was not detected. This demonstrates that, although lineage can be influenced by microenvironmental conditions, a strong lymphoid bias persists even when MLL-Af4-expressing cells have a myeloid cell phenotype (Fig. 3). This extreme degree of lineage plasticity is also seen in patients with MLL-AF4 infant leukaemia, where lineage switching can occur, particularly, upon CD19-targeted therapy (Park et al., 2011; Rayes et al., 2016; Wölfl et al., 2018).

Currently, the effect of aging on haematopoiesis is a particularly popular area of research. The impact of age appears to have an important influence on lineage determination in *MLL*-rearranged leukaemia. As previously discussed, *MLL*-rearrangements *in utero* can give rise to infant lymphoid, myeloid or mixed-lineage leukaemia. In adults, *MLL*-rearrangements are associated with lymphoid leukaemias but also occur in a wider range of myeloid disorders, such as myelodysplasia, therapy-related and *de novo* AML. These differences are well illustrated when considering the *MLL-AF9* fusion gene, as it causes B-ALL and AML in equal proportions in infants but AML exclusively in adults. Several leukaemia models support the hypothesis that age-related changes in the haematopoietic niche can influence the leukaemia lineage.

Rowe et al. have recently investigated the influence of age on leukaemia lineage selection in a murine model of *MLL*-rearranged leukaemia (Rowe et al., 2019). The group retrovirally transduced *MLL-AF9* and *MLL-ENL* into foetal liver LSK CD127^−^ HSPCs at mid-gestation. Both fusions are common in myeloid-lineage infant leukaemia, with *MLL-AF9* also associated with lymphoid-lineage infant leukaemia (Harrison et al., 2010; Hilden, 2006). Previous studies have shown that MLL-AF9-expressing HSPCs are only capable of transforming into a myeloid lineage leukaemia on transplantation (Lin et al., 2016; Milne, 2017). Rowe et al. transplanted the transduced HSPCs into neonatal and adult mice to assess whether age-related haematopoietic niche factors could influence leukaemia lineage (Rowe et al., 2019), and observed that this transplant caused a mixed myeloid/B-lymphoid leukaemia in neonatal mice, whereas adult mouse recipients developed a myeloid leukaemia (Fig. 3). In this model, the B-lymphoid differentiation seen in neonatal murine recipients with mixed-lineage leukaemia had an immunophenotype consistent with that of early pre-/pro-B differentiation. These lineage biases were maintained through serial transplantations. Interestingly, features of B-cell differentiation were seen in adult myeloid leukaemia cells that had been serially transplanted through neonatal recipients, suggesting a degree of continued niche-dependent lineage plasticity. No immunophenotypic differences in leukaemia lineage between neonatal and adult recipients was seen upon primary transplantation of *MLL-ENL*-transduced HSPCs; however, serial transplantation of neonatal MLL-ENL leukaemia cells through neonatal recipients did drive a mixed-lineage leukaemia with a mature B-cell component. Rowe and colleagues also presented RNA-sequencing data from these transplant studies, which showed the leukaemia that developed in neonatal recipients had upregulated expression of genes associated with early B-cell lineage commitment and a primitive HSPC state. Taken together, this evidence supports the role of age-dependent haematopoietic niche factors in lineage determination and, in particular, that the neonatal bone marrow niche supports mixed myeloid/B-lymphoid leukaemia differentiation (Rowe et al., 2019).

Chaudhury et al. have developed a model of AML using the *NUP98*-*HOXA9* (NH9) fusion oncogene to investigate how the age of the leukaemia-initiating cell may affect disease development (Chaudhury et al., 2018). LSK cells from murine foetal liver, as well as juvenile (3 weeks), adult (10 weeks) and aged (>52 weeks) murine bone marrow were transduced with NH9-expressing retrovirus, cultured *in vitro* and the resulting colonies assessed for leukaemic transformation and lineage identity – however, no significant differences in output between different cell ages were observed. Cells were then transplanted into adult mice to assess for *in vivo* development of leukaemia. Mice transplanted with ‘young’ cells – i.e. from foetal liver and juvenile bone marrow – developed AML, B-lymphoid or mixed-lineage leukaemia, whereas mice transplanted with adult and aged cells developed AML exclusively (Chaudhury et al., 2018). Previously, Horton et al. had made a similar observation regarding cell-of-origin age-dependent lineage fate decisions in a xenograft model of infant and adult MLL-AF9 acute leukaemia (Horton et al., 2013). The authors used human neonatal cord blood and adult CD34^+^ cells transduced with *MLL-AF9* and assessed their capacity for *in vitro* and *in vivo* leukaemic transformation. Transduced neonatal cells gave rise to both AML and ALL *in vivo*, whereas transduced adult cells resulted in long-term engraftment with a myeloid bias – but not in leukaemia (Horton et al., 2013).

The exploration by Chaudhury et al. regarding the distinction between *in vitro* and *in vivo* lineage determination led to investigation of the haematopoietic niche as a potential mediator of these differences. Their findings support an age-dependent bone marrow-remodelling process, and interactions between a leukaemia cell and the bone marrow niche that depend on the age of the transplanted cell. This model clearly contributes to our knowledge of niche-dependent processes in leukaemia development at different ages (Chaudhury et al., 2018).

### Placenta and central nervous system as other specialised niches

When considering the broadest definition of a cancer niche in infant leukaemia, there are two additional specialised niches: the placenta and the CNS.

During embryonic development, a substantial pool of HSCs appears in the placenta, concomitantly to the production of HSCs in the AGM. Placental HSCs expand until E12.5-E13.5 (Gekas et al., 2005). The placenta as an HSC niche is unique in its exposure to, and possible influence from, exogeneous signalling through its main function as the foetal-maternal interface. On the basis of the Mll-AF4^+^ VEC-Cre^+^ pre-leukaemic mouse model we discussed earlier in this Review, our group developed a model system to investigate whether inflammatory signals of maternal origin provide the necessary trigger to cause full leukaemia transformation (Malouf and Ottersbach, 2019). This hypothesis was supported by evidence found in non-infant paediatric ALL, where infections can lead to key secondary mutations that facilitate leukaemogenesis, and has recently been reviewed by Mel Greaves (Greaves, 2018). We tested the effects of inflammatory signals *in vitro* and *in vivo*, by harvesting LSK cells from foetal liver during the pre-leukaemic phase, and exposing them to viral- and bacterial-like immune stimuli. Cells that had received immune stimulation showed increased proliferation but not clonogenic potential. We then injected the viral mimic polyinosinic:polycytidylic acid [poly(I:C)] into pregnant dams at the same time point as in the *in vitro* assay. This treatment, indeed, induced an appropriate immune activation in the pregnant dams but was insufficient to cause leukaemic transformation in the resulting pups (Malouf and Ottersbach, 2019).

**Fig. 4.**
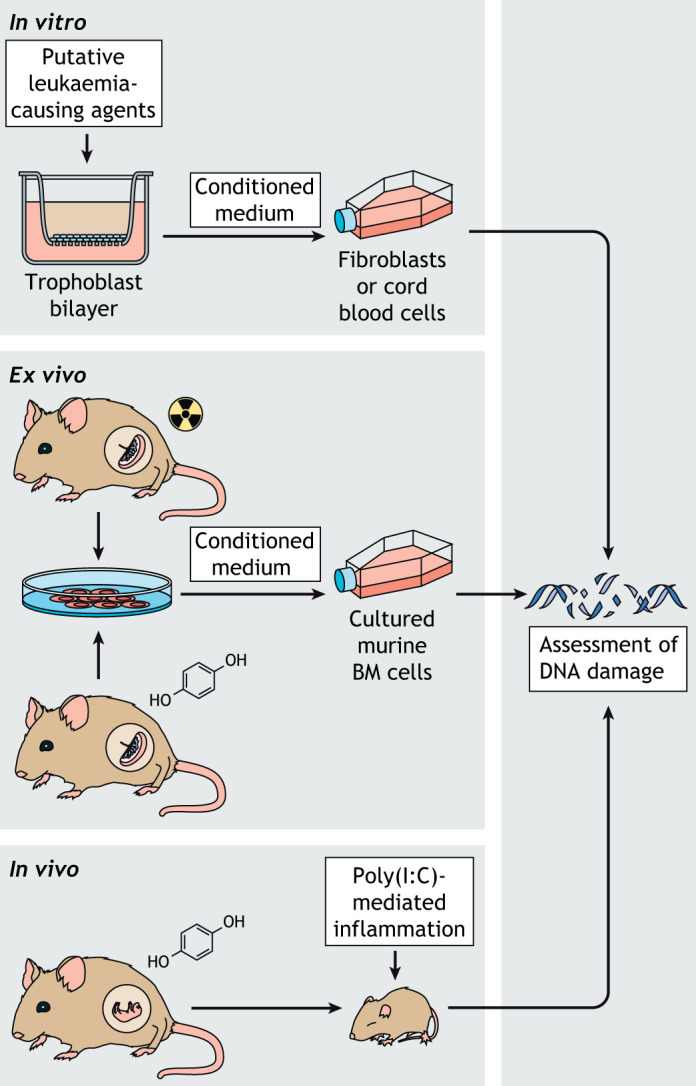
**Mouse models developed by Mansell et al. (2019).** These mouse models were designed to investigate haematopoietic niche factors that initiate or promote malignant transformation, which can be assessed by measuring DNA damage. (Top) The *in vitro* model of the trophoblast barrier exposes a bilayer of cultured trophoblast cells to putative leukaemia-causing agents, including chromium, poly(I:C), LPS, hypoxia and etoposide. The collected ‘conditioned medium’ is then used in further cell culture of fibroblasts and human cord blood cells, and any DNA damage to these cells is measured. (Middle) The *ex vivo* model involves exposing pregnant dams at E12 to radiation or the benzene metabolite hydroquinone. The harvested placental cells are then cultured and the ‘conditioned medium’ is used to culture mouse bone marrow (BM) cells, which are then assessed for DNA damage. (Bottom) The *in vivo* model involves keeping the offspring of pregnant dams following hydroquinone exposure at E12 and then attempting to initiate a second mutational hit in the pups through poly(I:C)-induced inflammation. LPS, lipopolysaccharide; poly(I:C), polyinosinic:polycytidylic acid.

Mansell et al. also considered whether the placental niche, through its exposure to environmental factors, could initiate leukaemia (Mansell et al., 2019). For this, they focused on developing models to investigate the role of the placental barrier, which separates maternal and foetal blood, and whether signals from these placental trophoblast cells can induce leukaemia-initiating mutations in foetal HSPCs through a so-called bystander effect. The three model systems they developed – an *in vitro*, *ex vivo* and *in vivo* model – are summarised in Fig. 4. In the *in vitro* model, DNA damage is present in both fibroblasts and cord blood cells upon indirect exposure to agents with strong evidence of leukaemogenic potential – including pesticides, benzene and radiation – through an unknown DNA-damaging factor that is present in the conditioned medium. In some cases, the DNA damage is associated with cytogenetic abnormalities seen in paediatric leukaemia, such as hypodiploidy. In the *ex vivo* model, bone marrow cells grown in conditioned medium from irradiated placental cultures show increased DNA damage, demonstrating *in vivo* production of DNA-damaging factors by the placenta in response to radiation, to which bone marrow cells are sensitive. They also demonstrated that DNA damage induced by exposure to the benzene metabolite hydroquinone can be prevented by treating the pregnant dams with the antioxidant mitoquinol. In the *in vivo* model, DNA damage, including double-strand breaks, is present in bone marrow cells derived from pups exposed to both indirect intrauterine hydroquinone and direct postnatal poly(I:C) treatment. In these double-treated pups, DNA damage was specifically identified when purified HSCs were cultured under conditions that promote inflammation-mediated HSC proliferation. Again, they found that treatment of the pregnant dams with mitoquinol prevents DNA damage. They concluded, therefore, that indirect intrauterine hydroquinone exposure mimics the ‘first hit’ in the two-hit paediatric leukaemia hypothesis, with resurgence of the damage following an inflammatory stimulus, thus, representing the second hit, as postulated in the two-hit model by Mel Greaves (Greaves, 2018). These three mouse models demonstrate that – after indirect exposure to cancer causing substances via maternal circulation – the placenta is a site of mutation acquisition in HSCs. This niche-specific feature may support the oncogenic development in infant and paediatric leukaemia.

An important clinical feature of infant leukaemia is the high involvement of the CNS at diagnosis and relapse (Hilden, 2006; Pieters et al., 2019). Indeed, any truly representative model of infant leukaemia should demonstrate the typical leptomeningeal leukaemic infiltrate seen in patients (Price and Johnson, 1973). The above described Mll-AF4^+^ VEC-Cre^+^ miR-128^+^ and/or miR-130b^+^ overexpression models of infant leukaemia (Malouf et al., 2021) demonstrate this typical CNS disease pattern. Both miRNAs drive different lineages of leukaemia, with leptomeningeal leukaemia infiltrate present to a more significant extent in the pro-B ALL driven by miR-128a overexpression compared with the mixed/B-cell precursor/myeloid lineage acute leukaemia driven by miR-130b overexpression. Investigating the CNS niche in infant leukaemia is particularly compelling because there are so many unknowns, including why infant leukaemia has such a strong predisposition for this site and how leukaemia cells enter, survive and propagate in the niche.

In terms of nutrient and metabolite supply as well as cellular composition, the leptomeningeal niche is in stark contrast to the primary leukaemia sites, i.e. the BM and blood. This has been highlighted by Savino et al., who found that the fatty acid content of both murine and human cerebrospinal fluid (CSF), which bathes the leptomeningeal structures, is particularly low compared to that of blood plasma. By using a murine xenograft model of paediatric ALL, the group demonstrated that leukaemia cells in the leptomeningeal niche undergo metabolic rewiring through the upregulation of genes involved in *de novo* fatty acid synthesis, such as stearoyl-CoA desaturase and fatty acid synthase, to overcome this critical lack of environmentally available fatty acids. The leukaemia-initiating cells used in this xenograft model were from an MLL-AF4 ALL cell line (SEM) and, although this cell line was derived from a 6-year-old patient, the findings are likely to be similar in infant leukaemia given the shared genetic basis and the fact that CNS involvement is a particularly prominent complication in infant patients. This indicates that crucial niche-dependent metabolic adaptations are required for leukaemia cell survival in the leptomeningeal niche (Savino et al., 2020).

Little is known of the non-leukaemic cellular composition of the CNS niche in either infant or paediatric ALL. In health, the immune composition of the meninges is complex, with dynamic changes that occur during embryonic development and aging. During embryonic development, extra-parenchymal CNS macrophage populations, including tissue-resident leptomeningeal macrophages, are established from yolk sac erythro-myeloid progenitors (Goldmann et al., 2016). These populations appear to have self-renewal capacity but they are also replenished from the adjacent skull bone marrow. Importantly, leptomeningeal macrophages are not replenished from circulating blood-derived cells. The complexity of the myeloid immune landscape in the meninges is mirrored in the lymphoid contribution, where meningeal B cells derived from skull bone marrow display age-dependent maturation profiles (Brioschi et al., 2021). These developmentally dependent features of the immune cell profile in the meninges may influence the propensity of leukaemias to thrive in the leptomeningeal niche.

There are many hypothesised routes of leukaemia cell entry into the leptomeninges, one of which involves cancer cells crossing into the CSF from the systemic circulation across the choroid plexus epithelium (Frishman-Levy and Izraeli, 2017). The choroid plexus is responsible for CSF production and a key element of the blood-CSF barrier. Fernández-Sevilla et al. recently showed that ALL cells infiltrate the choroid plexus in a murine xenograft model (Fernández-Sevilla et al., 2020). They demonstrated that B-cell precursor ALL cells and human choroid plexus fibroblasts interact *in vitro*, mediated by reciprocal expression of adhesion molecules, and that choroid plexus fibroblasts acquire a cancer-associated fibroblast phenotype. Moreover, co-culture with choroid plexus fibroblasts protected the leukaemic cells when exposed to methotrexate or cytarabine, drugs commonly used in treating CNS leukaemia in infant and paediatric patients. Overall, these findings suggest that the choroid plexus, specifically the fibroblasts within this structure, forms part of the CNS leukaemia niche and is important in facilitating leukaemia cell survival at this site (Fernández-Sevilla et al., 2020). In particular, the study scratches the surface of two important considerations. First, given the particularly high rates of CNS involvement in infants at presentation and relapse, are there infant-specific features of the CNS niche that cooperate with leukaemia development? Second, can the anti-leukaemic efficacy of current therapies be improved by disrupting these interactions?

## Conclusions

The evaluation of *in vitro* and *in vivo* models of infant and paediatric leukaemia, in which the haematopoietic niche has been a focus of analysis, proves that niche-dependent factors have significant impact on leukaemia initiation and lineage determination. Table 1 compares the haematopoietic niches involved in developing and sustaining infant leukaemia. However, our understanding of the underlying biology is still elusive. Our knowledge of the molecular mechanisms involved in normal HSPC-niche interactions during developmental haematopoiesis and in leukaemia initiation during this stage requires ongoing research attention.

**Table 1.**
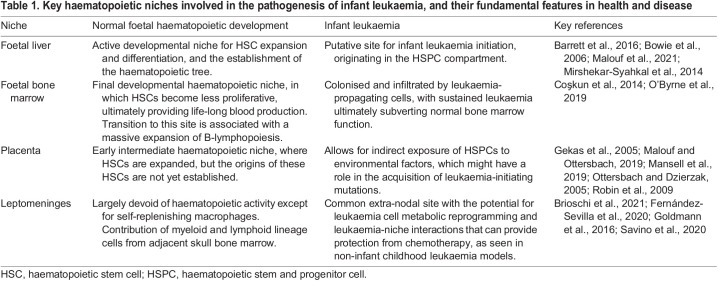
Key haematopoietic niches involved in the pathogenesis of infant leukaemia, and their fundamental features in health and disease

Further research in this area is particularly important owing to its potential to develop new therapeutics, particularly as outcomes for infant leukaemia trail those seen in paediatric leukaemias. The need for new therapies benefitting infant leukaemia is particularly strong because improvements through iterative changes to conventional chemotherapy regimens are lacking.

In addition, treatment-related mortality associated with current therapy protocols is high, especially when involving stem cell transplantation, e.g. 14.4% in the INTERFANT-06 trial (Pieters et al., 2019). Understanding the unique biology of infant leukaemia imposed by its foetal origin may highlight specific vulnerabilities that can be exploited to establish more-targeted therapies. Furthermore, harnessing the leukaemia niche as a therapy is a realistic option. By targeting leukaemia niche-specific interactions, selectively ridding the niche of leukaemia stem cells while sparing normal HSCs is, theoretically, achievable. The development of novel, more-targeted treatments, to reduce the reliance on intensive chemotherapy and transplants, is desperately needed to improve long-term survival for these patients and to reduce current therapy-related long-term health issues.
